# Hsa_circ_0110757 upregulates ITGA1 to facilitate temozolomide resistance in glioma by suppressing hsa-miR-1298-5p

**DOI:** 10.1038/s41419-021-03533-x

**Published:** 2021-03-05

**Authors:** Haoyu Li, Qing Liu, Zihua Chen, Ming Wu, Chao Zhang, Jun Su, Yue Li, Chi Zhang

**Affiliations:** 1 Department of Neurosurgery, Xiangya Hospital, Central South University, Changsha, 410008 China; 2 The Institute of Skull Base Surgery and Neurooncology at Hunan Province, Changsha, 410008 China; 3 Hunan Key Laboratory of Precise Diagnosis and Treatment of Gastrointestinal Tumor, Changsha, 410008 China; 4 Department of General Surgery, Xiangya Hospital, Central South University, Changsha, 410008 China

**Keywords:** Chemotherapy, Tumour-suppressor proteins

## Abstract

Temozolomide (TMZ) is the internationally recognized and preferred drug for glioma chemotherapy treatment. However, TMZ resistance in glioma appears after long-term use and is an urgent problem that needs to be solved. Circular RNAs (circRNAs) are noncoding RNAs and play an important role in the pathogenesis and progression of tumors. Hsa_circ_0110757 was identified in TMZ-resistant glioma cells by high-throughput sequencing analysis and was derived from reverse splicing of myeloid cell leukemia-1 (Mcl-1) exons. The role of hsa_circ_0110757 in TMZ-resistant glioma was evaluated both in vitro and in vivo. It was found that hsa_circ_0110757 and ITGA1 are more highly expressed in TMZ-resistant glioma than in TMZ-sensitive glioma. The overexpression of hsa_circ_0110757 in glioma patients treated with TMZ was obviously associated with tumor invasion. This study indicates that hsa_circ_0110757 inhibits glioma cell apoptosis by sponging hsa-miR-1298-5p to promote ITGA1 expression. Thus, hsa_circ_0110757/hsa-miR-1298-5p/ITGA could be a potential therapeutic target for reversing the resistance of glioma to TMZ.

## Introduction

Glioma, originating from the neuroepithelium and accounting for 40–50% of intracranial tumors, is the most common malignant tumor of the central nervous system^1,2^. The main characteristics are diffuse invasive growth of tumor cells, unclear boundaries, relatively unlimited proliferation, and high invasiveness, all of which seriously affect human health^3^. Despite advances in the treatment of gliomas, the 5-year survival rate of glioma patients remains low^4^. For advanced glioma patients, systemic chemotherapy is main form of treatment^5^. However, drug resistance is still an important cause of chemotherapy failure in many cancers including glioma^6,7^. Therefore, it is an urgent problem to overcome the chemotherapy drug resistance in glioma.

Currently, temozolomide (TMZ) is the internationally recognized and preferred drug for glioma chemotherapy^8,9^. TMZ is the second generation of alkalization agent that can be widely distributed throughout the body without liver metabolism and can enter the brain through the blood–brain barrier (BBB), achieving high drug concentrations in the brain^10,11^. However, long-term clinical research has found that TMZ can only prolong survival time to a small extent, and a considerable number of gliomas are still insensitive to TMZ and gradually develop drug resistance^12^. Therefore, the resistance of glioma to TMZ is considered to be the fundamental cause of chemotherapy failure and glioma recurrence^13^.

Circular RNAs (circRNAs) are noncoding RNAs that can promote or inhibit tumorigenesis^14^. circRNAs competitively inhibit the expression of endogenous RNAs by sponging microRNAs (miRNAs), which is a novel mechanism of regulating miRNA expression^15,16^. Due to many biological processes adjusted by miRNAs, circRNAs can alter biological processes by sponging miRNAs^17^. miRNAs are a ubiquitous kind of short noncoding RNA (~22 nt) that can be directly paired with target bases in the mRNA and regulate gene expression after transcription^18^. circRNAs can influence miRNA function by competitively binding to miRNA sites^19^. Nevertheless, the role of circRNAs as miRNA sponges in TMZ-resistant glioma has not been fully clarified.

To explore the regulatory effect of circRNAs on TMZ-resistant glioma, high-throughput sequencing was performed, and there were many different circRNAs in TMZ-resistant and TMZ-sensitive glioma tissues. Through many experiments, we found that hsa_circ_0110757, originating from myeloid cell leukemia-1 (Mcl-1) exons, was obviously overexpressed in TMZ-resistant and TMZ-sensitive glioma tissues and cells. In addition, we found that hsa_circ_0110757 facilitates TMZ resistance modulation by sponging hsa-miR-1298-5p, which inhibits Integrin subunit alpha 1 (ITGA1) expression by activating the PI3K/AKT pathway in glioma.

## Results

### Hsa_circ_0110757 expression level in TMZ-resistant glioma tissues and cells

**Fig. 1:**
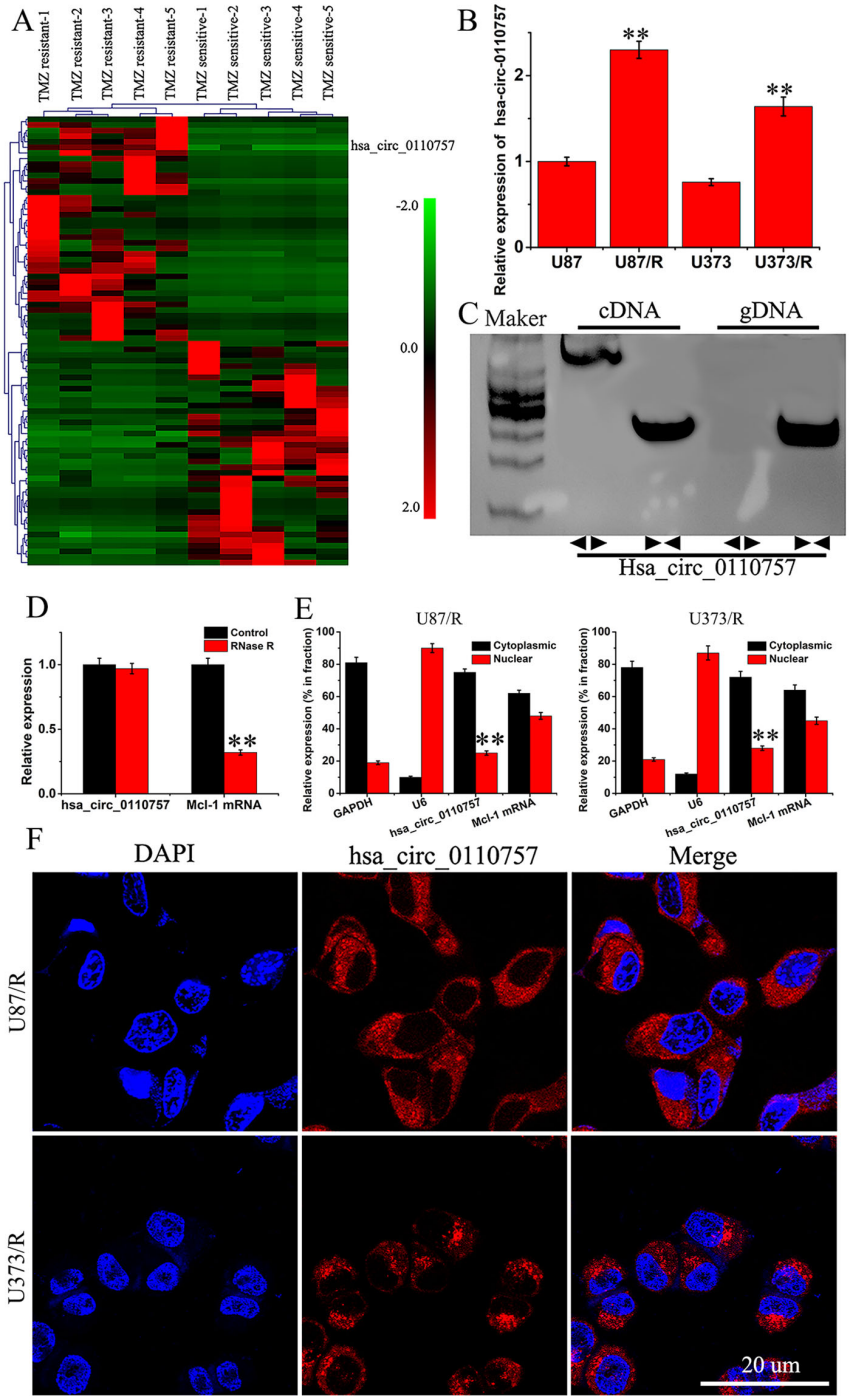
The expression of hsa_circ_0110757 in TMZ-resistant glioma tissues and cells. **A** The circRNA expression profile in five TMZ-resistant glioma tissues and five TMZ-sensitive glioma tissues by high-throughput sequencing. **B** The expression of hsa_circ_0110757 in U87, U87/R, U373, and U373/R cells. **C** Hsa_circ_0110757 was verified in U87/R by RT-PCR. hsa_circ_0110757 was amplified by divergent primers in cDNA but not in gDNA. **D** The stability of hsa_circ_0110757 and Mcl-1 RNA after enzymolysis with or without RNase R. **E** The distribution of hsa_circ_0110757 and Mcl-1 RNA in the nuclear and cytoplasmic fractions of U87/R and U373/R cells. U6 was used for nuclear fraction positive control, GAPDH was used for cytoplasmic fraction positive control. **F** The distribution of hsa_circ_0110757 was detected by fluorescence in situ hybridization (FISH) in U87/R and U373/R cells. **P < 0.01.

RNA-Seq assays were conducted to identify differentially expressed circRNAs in TMZ-sensitive patients and TMZ-resistant patients. As shown in Fig. 1A, various circRNAs were differentially expressed in TMZ-resistant patients. Among the obviously upregulated circRNAs, RT-qPCR was used to verify their expression levels. Hsa_circ_0110757 was the most significantly overexpressed circRNA in TMZ-resistant glioma tissues, which was derived from the reverse splicing of Mcl-1 exons. Subsequently, TMZ-resistant glioma cells (U87/R and U373/R) were established. The IC50 of TMZ in U87, U87/R, U373, and U373/R cells were 5.3, 18.5, 7.1, and 19.2 μmol/l, respectively (Fig. S1). In agreement with the RNA-Seq results, hsa_circ_0110757 was obviously increased in U87/R cells (Fig. 1B). To exclude the existence of genomic rearrangements or trans-splicing, convergent primers for Mcl-1 mRNA and divergent primers for hsa_circ_0110757 were designed. Hsa_circ_0110757 could only be amplified in cDNA but not in gDNA of U87/R cells by divergent primers (Fig. 1C). Furthermore, linear Mcl-1 mRNA was digested, but hsa_circ_0110757 remained intact after RNase R enzymolysis (Fig. 1D). Next, the expression of hsa_circ_0110757 was determined in both the cytoplasm and nucleus of U87/R cells, which showed that hsa_circ_0110757 was mainly enriched in the cytoplasm (Fig. 1E). In addition, the FISH results showed that hsa_circ_0110757 was mainly distributed in the cytoplasm (Fig. 1F).

### Hsa_circ_0110757 induces TMZ resistance in vitro

**Fig. 2:**
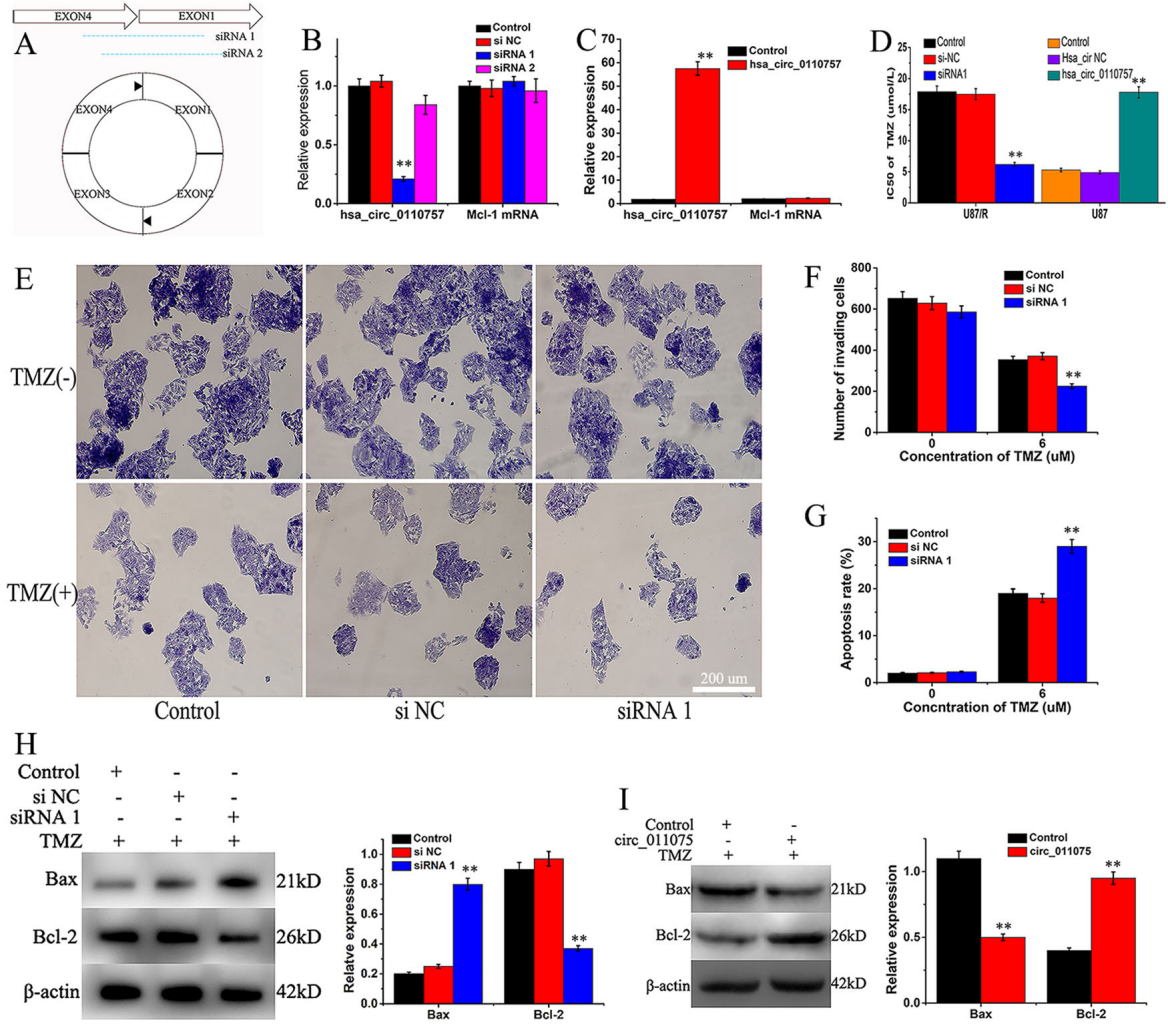
Hsa_circ_0110757 promotes TMZ resistance of glioma cell in vitro. **A** The siRNA targeting the back-splice junction (siRNA 1 and siRNA 2). **B** The expression of hsa_circ_0110757 and Mcl-1 mRNA in U87/R cells with or without siRNA by RT-PCR. **C** The expression of hsa_circ_0110757 and Mcl-1 mRNA in U87 cells with or without stable transfection of hsa_circ_0110757 by RT-PCR. U87 cells without any transfection were set as control group. **D** The IC50 of TMZ in U87/R cells with transfection of si-hsa_circ_0110757 and U87 cells with transfection of hsa_circ_0110757 for 48 h. **E**, **F** The invasion ability of the U87/R cells. **G** The apoptotic rates of U87/R cells with or without siRNA 1 in the absence or presence of TMZ. **H** The expression of Bax and Bcl-2 in U87/R cells with or without siRNA 1 in the presence of TMZ for 24 h. **I** The expression of Bax and Bcl-2 in U87/R cells with or without hsa_circ_0110757 in the presence of TMZ for 24 h. *P < 0.05, **P < 0.01, ***P < 0.001.

First, two siRNAs targeting hsa_circ_0110757 were constructed (Fig. 2A). Hsa_circ_011075 but not Mcl-1 expression was successfully knocked down by siRNA 1 in U87/R cells (Fig. 2B). The specificity of siRNA on hsa_circ_0110757 was also validated in hsa_circ_0110757 overexpressed U87 cells. As shown in Fig. S2, hsa_circ_011075 was successfully knocked down by siRNA 1 rather than siRNA 2. To evaluate the role of hsa_circ_0110757, hsa_circ_0110757 was upregulated in U87 cells via adenovirus transfection and had no effect on Mcl-1 mRNA (Fig. 2C). High expression of hsa_circ_0110757 caused U87 cells to develop resistance to TMZ. However, inhibition of hsa_circ_0110757 restored sensitivity of U87/R cells to TMZ (Fig. 2D), reduced the number of invading cells (Fig. 2E, F), and promoted cell apoptosis (Fig. 2G). The underlying mechanism of cell viability was investigated by western blotting. When TMZ was applied, expression of the proapoptotic protein Bax was increased, and expression of antiapoptosis protein Bcl-2 was decreased in hsa_circ_011075-knockdown U87/R cells (Fig. 2H). In contrast, the inverse protein levels were discovered when hsa_circ_0110757 was upregulated (Fig. 2I).

### hsa_circ_0110757 works by sponging hsa-miR-1298-5p

**Fig. 3:**
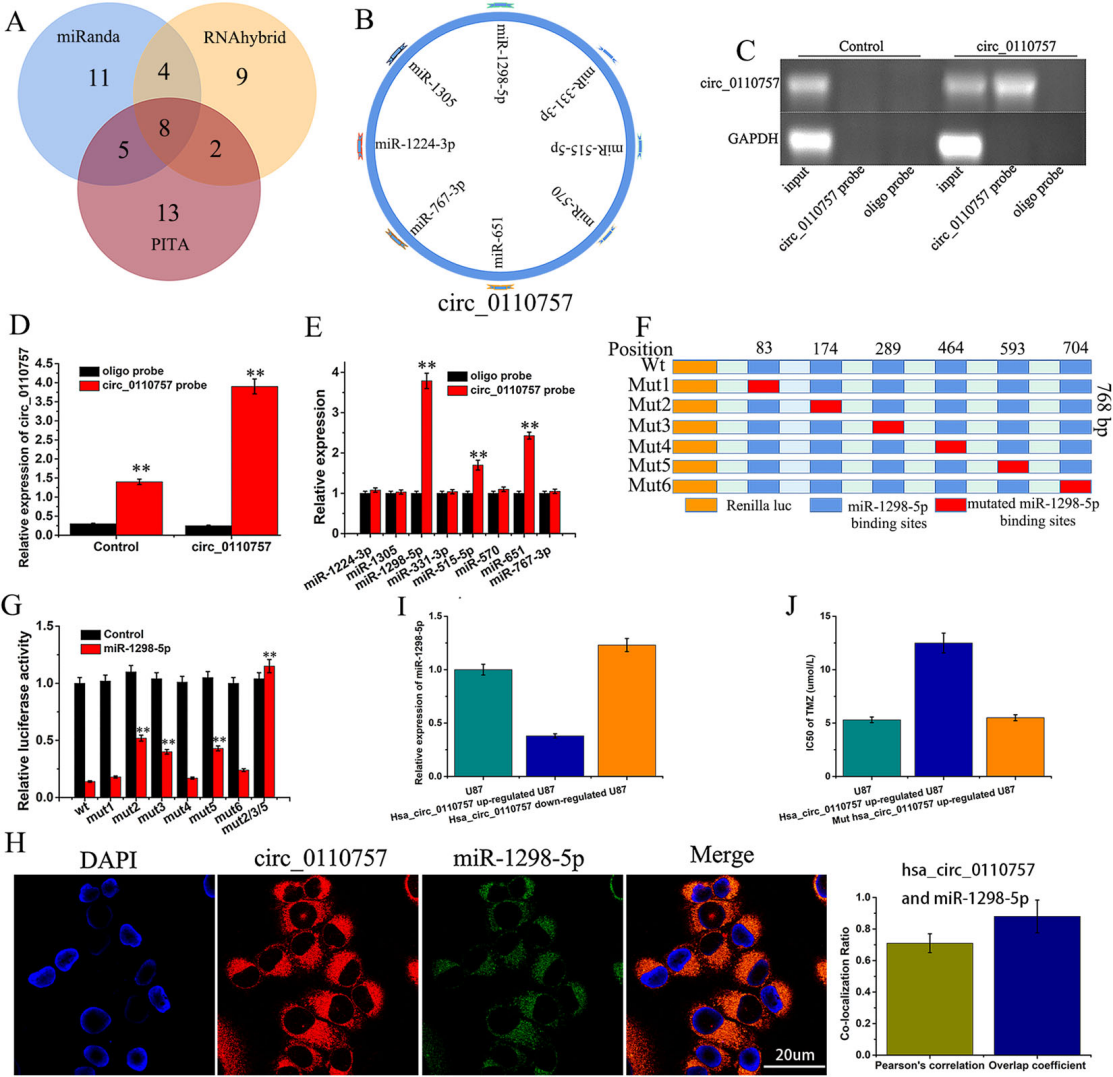
Hsa_circ_0110757 works by sponging hsa-miR-1298-5p. **A**, **B** The target miRNAs of hsa_circ_0110757 were deduced by miRanda, PITA, and RNAhybrid. **C**, **D** Lysates from U87/R cells with hsa_circ_0110757 transfection were determined by PCR after RNA pull-down assay. **E** The expression of eight miRNAs pulled down by hsa_circ_0110757 in U87/R cells were determined by RT-qPCR. **F** The schematic illustration of luciferase reporters containing wild-type hsa_circ_0110757 (wt) or the hsa_circ_0110757 with mutated miR-1298-5p (mut1–mut6) binding sites. **G** The luciferase activity of wt and mut1–mut6 in U87 cells cotransfected with miR-1298-5p. **H** The colocalization ratio of hsa_circ_0110757 and miR-1298-5p in U87/R cells by FISH. **I** The expression of miR-1298-5p in hsa_circ_0110757 overexpressed or repressed in U87 cells. **J** The IC50 of TMZ in wild and mutated hsa_circ_0110757 transfected U87 cells. **P < 0.01.

To identify miRNAs that hsa_circ_0110757 could sponge in glioma, eight miRNAs were chosen by overlapping the prediction results using miRanda, PITA, and RNAhybrid (Fig. 3A, B). A pull-down assay was used to detect whether the eight miRNAs could bind hsa_circ_0110757. A biotin-linked hsa_circ_0110757 probe was designed to pull down hsa_circ_0110757 in U87/R cells, and the pull-down effectiveness was obviously elevated in cells overexpressing hsa_circ_0110757 (Fig. 3C, D). After pull down, the eight miRNAs were extracted and detected by PCR. As shown in Fig. 3E, hsa-miR-1298-5p was significantly pulled down by hsa_circ_0110757 in U87/R cells. In the luciferase reporter assays, hsa-miR-1298-5p obviously decreased the luciferase activity of the wild-type (wt) hsa_circ_0110757 sequence (wt) compared to the luciferase activity of hsa_circ_0110757 with mutated hsa-miR-1298-5p binding sites (mut2, mut3, and mut5) (Fig. 3F, G). Hsa_circ_0110757 and hsa-miR-1298-5p were colocalized in the cytoplasm according to a FISH assay (Fig. 3H). Furthermore, the expression level of miR-1298-5p in U87/R cells and TMZ-resistant patients were much lower than that of U87 cells and TMZ-sensitive patients (Fig. S3). In addition, as shown in Fig. 3I, hsa-miR-1298-5p was downregulated in hsa_circ_0110757 overexpressed U87 cells and upregulated in hsa_circ_0110757 low expressed U87 cells, which demonstrated that hsa_circ_0110757 does indeed suppress hsa-miR-1298-5p. In order to test whether hsa_circ_0110757 has specific effect on acquiring resistance to TMZ, wild and mutated hsa_circ_0110757 were transfected in U87 cells. As shown in Fig. 3J, High expression of wild hsa_circ_0110757 but not mutated hsa_circ_0110757 induces U87 cells to acquire resistance to TMZ.

### ITGA1 is a regulatory target of hsa-miR-1298-5p

**Fig. 4:**
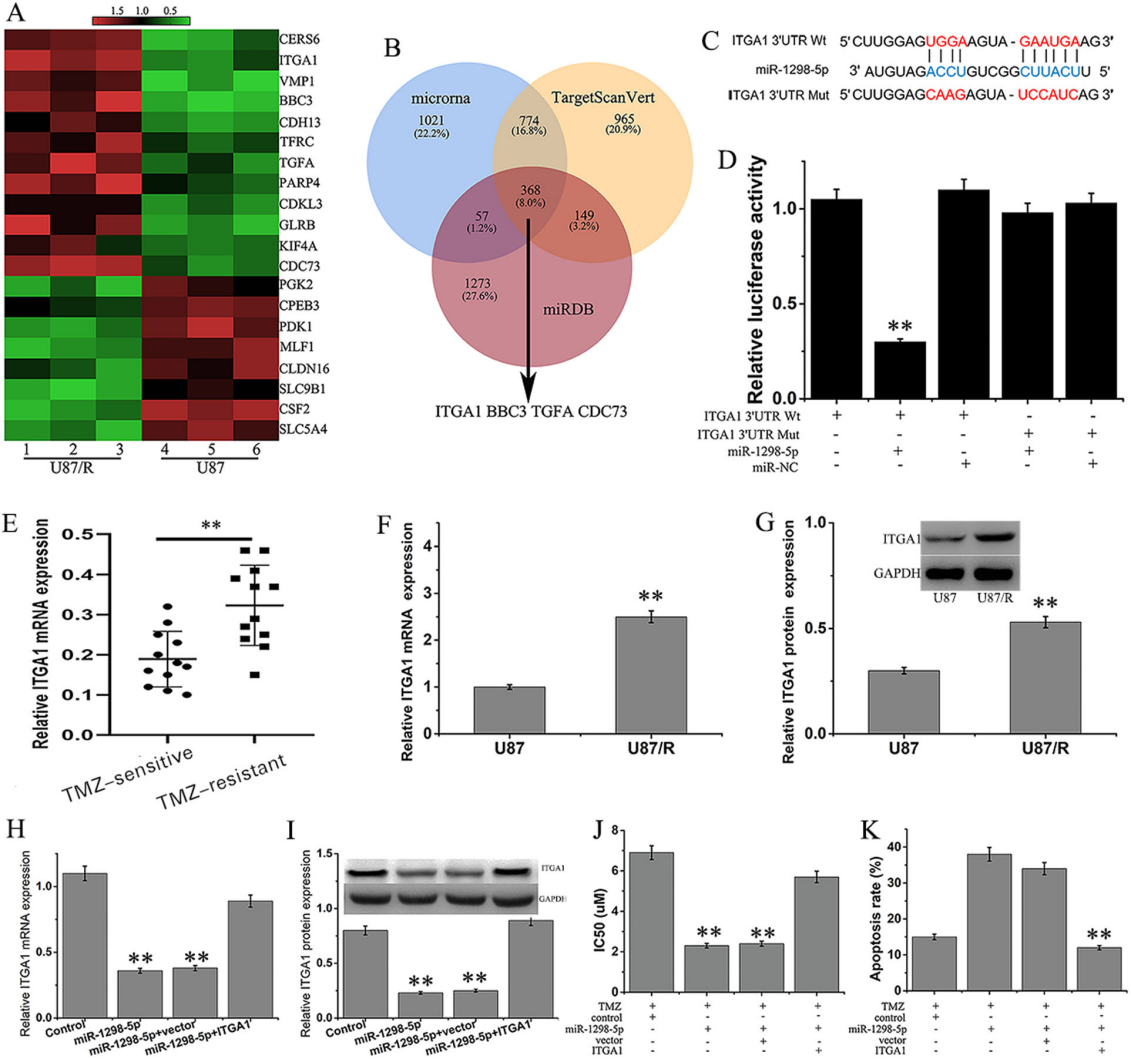
ITGA1 is a regulatory target of hsa-miR-1298-5p. **A** The differential expressed genes in U87/R and U87 cells (*n* = 3) by high-throughput sequencing. **B** Four genes are hsa-miR-1298-5p targets predicted by miRanda, miRDB, and TargetScan. **C** The pattern of ITGA1 3′ UTR wild-type (WT) and mutated type (Mut) luciferase reporter. **D** Relative activity of luciferase in U87 cells with miR-1298-5p mimics and ITGA1(Wt) or ITGA1 (Mut). The expression of ITGA1 in TMZ-sensitive, TMZ-resistant tissues (**E**) and U87, U87/R cells (**F**) was detected by RT-qPCR. **G** ITGA1 protein was determined by western blot in U87 and U87/R cells. ITGA1 was detected by RT-qPCR (**H**) and western blot (**I**). U87/R cells were cotransfected with miR-1298-5p mimic or ITGA1. The IC50 of TMZ (**J**) and apoptosis rates (**K**) in U87/R cells cotransfected with miR-1298-5p mimic alone or ITGA1. **P < 0.01.

Further high-throughput sequencing was performed on U87 and U87/R cells to identify the differentially expressed genes (Fig. 4A). The top 12 upregulated genes indentified by high-throughput sequencing were analyzed by three algorithms (microRNA, TargetScanVert, and miRDB) prediction, the 3′ UTRs of ITGA1, BBC3, TGFA, and CDC73 could be targets of hsa-miR-1298-5p (Fig. 4B). Whether hsa-miR-1298-5p can directly target these genes in U87 cells was determined by luciferase reporter assays (Fig. 4C, D, Fig. S4). In U87 cells transfected with hsa-miR-1298-5p, the luciferase activity of a reporter with wt hsa-miR-1298-5p binding sites in ITGA1 was reduced compared with that with mutated binding sites (Fig. 4D). As a result, hsa-miR-1298-5p can directly target ITGA1. ITGA1, also known as CD49a or VLA1, plays an important role in cell–cell adhesion. ITGA1 was upregulated in TMZ-resistant pancreatic cancer, and downregulated ITGA1 restored the sensitivity of the above cells to TMZ[24]. Compared with TMZ-sensitive tissues, the mRNA of ITGA1 in TMZ-resistant tissues was obviously increased (Fig. 4E). Consistent with previous results, ITGA1 mRNA and protein levels were much higher in U87/R cells than that in U87 cells (Fig. 4F, G). In addition, hsa-miR-1298-5p mimics obviously decreased the mRNA and protein of ITGA1, and ectopic ITGA1 expression offset the effect of overexpressed hsa-miR-1298-5p (Fig. 4H, I). Furthermore, overexpressed hsa-miR-1298-5p reduced cell viability and induced apoptosis in U87/R cells. However, co-overexpression of ITGA1 and hsa-miR-1298-5p abrogated apoptosis (Fig. 4J, K).

### Hsa_circ_0110757 promotes TMZ resistance by the ITGA1/PI3k/AKT/Bcl-2 signaling pathway

**Fig. 5:**
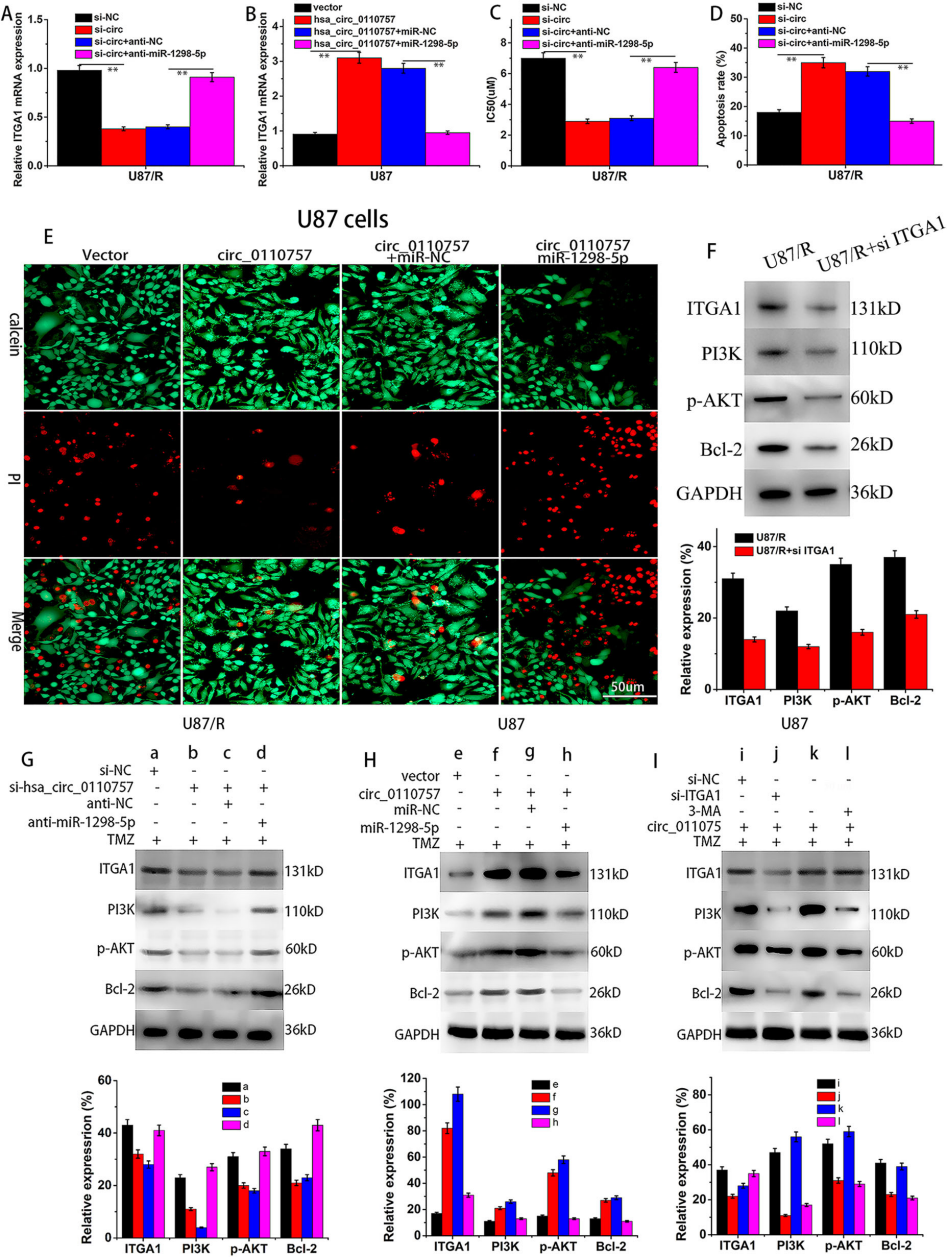
Hsa_circ_0110757 promotes TMZ resistance by ITGA1/PI3k/AKT/Bcl-2 signaling pathway. The expression of ITGA1 were determined by RT-qPCR. **A** Control vectors or inhibitors were cotransfected in U87/R cells. **B** U87 cells were cotransfected with control vectors and miR-1298-5p. **C** IC50 of TMZ was detected in U87/R cells transfected with the indicated vectors with TMZ application. **D** The apoptosis rates of U87/R cells with different transfection. **E** Live/dead cell staining of U87 cells with indicated transfection. Live cells were stained with green fluorescent, and dead cells were stained with red fluorescent. **F** The expression levels of ITGA1, PI3K/AKT, and antiapoptotic protein Bcl-2 were detected by western blotting in U87/R cells transfected with si-ITGA1. The expression levels of ITGA1, PI3K/AKT, and antiapoptotic protein Bcl-2 were detected by western blotting in U87/R cells transfected with indicated vectors alone or cotransfected with the inhibitor (**G**), in U87 cells transfected with the indicated vectors and miR-1298-5p (**H**) and in U87 cells transfected with the indicated vectors and 3-MA (**I**). **P < 0.01.

Anti-hsa-miR-1298-5p offset the downregulation of ITGA1 induced by si-hsa_circ_0110757 in U87/R cells (Fig. 5A). Hsa_circ_0110757 and hsa-miR-1298-5p cotransfection reduced ITGA1 expression compared to hsa_circ_0110757 transfection in U87 cells (Fig. 5B). In the cell viability and flow cytometry assay, si-hsa_circ_0110757 reduced cell viability and promoted apoptosis with TMZ application. However, si-hsa_circ_0110757 and anti-hsa-miR-1298-5p cotransfection obviously increased cell viability and suppressed apoptosis (Fig. 5C, D). In addition, overexpressed hsa_circ_0110757 inhibited cell apoptosis. However, hsa_circ_0110757 and miR-1298-5p cotransfection promoted cell apoptosis (Fig. 5E). In order to explore the mechanistic link between ITGA1 expression and activation of PI3K/AKT, ITGA1 was knocked down in U87/R cells. As shown in Fig. 5F, si-ITGA1 suppressed the activation of PI3k/AKT, which indicated that overexpressed ITGA1 induced the activation of PI3k/AKT in U87/R cells. si-hsa_circ_0110757 obviously decreased ITGA1 expression and suppressed the activation of PI3k/AKT, and downregulation of hsa_circ_0110757 and miR-1298-5p abolished these effects in U87/R cells (Fig. 5G). Overexpressed hsa_circ_0110757 obviously elevated the expression of the ITGA1 and PI3K/AKT signaling molecules, and cotransfection of hsa_circ_0110757 and miR-1298-5p abolished these effects in U87 cells (Fig. 5H). si-ITGA1 notably suppressed the expression of the ITGA1 and PI3K/AKT signaling molecules in U87 cells overexpressing hsa_circ_0110757. Next, 3-methyladenine (3-MA, a PI3K inhibitor) was used to explore whether the inactivation of PI3K/AKT can overwhelm the changes induced by overexpressed hsa_circ_0110757. The western blot results showed that si-ITGA1 and 3-MA notably suppressed the expression of ITGA1 and PI3K, and reduced the expression of p-AKT and Bcl-2 (Fig. 5I). The above results indicated that hsa_circ_0110757 works by sponging miR-1298-5p to promote the expression of ITGA1, and by activating PI3K/AKT signaling to induce TMZ resistance.

### Hsa_circ_0110757 facilitates TMZ resistance in glioma cells in vivo

**Fig. 6:**
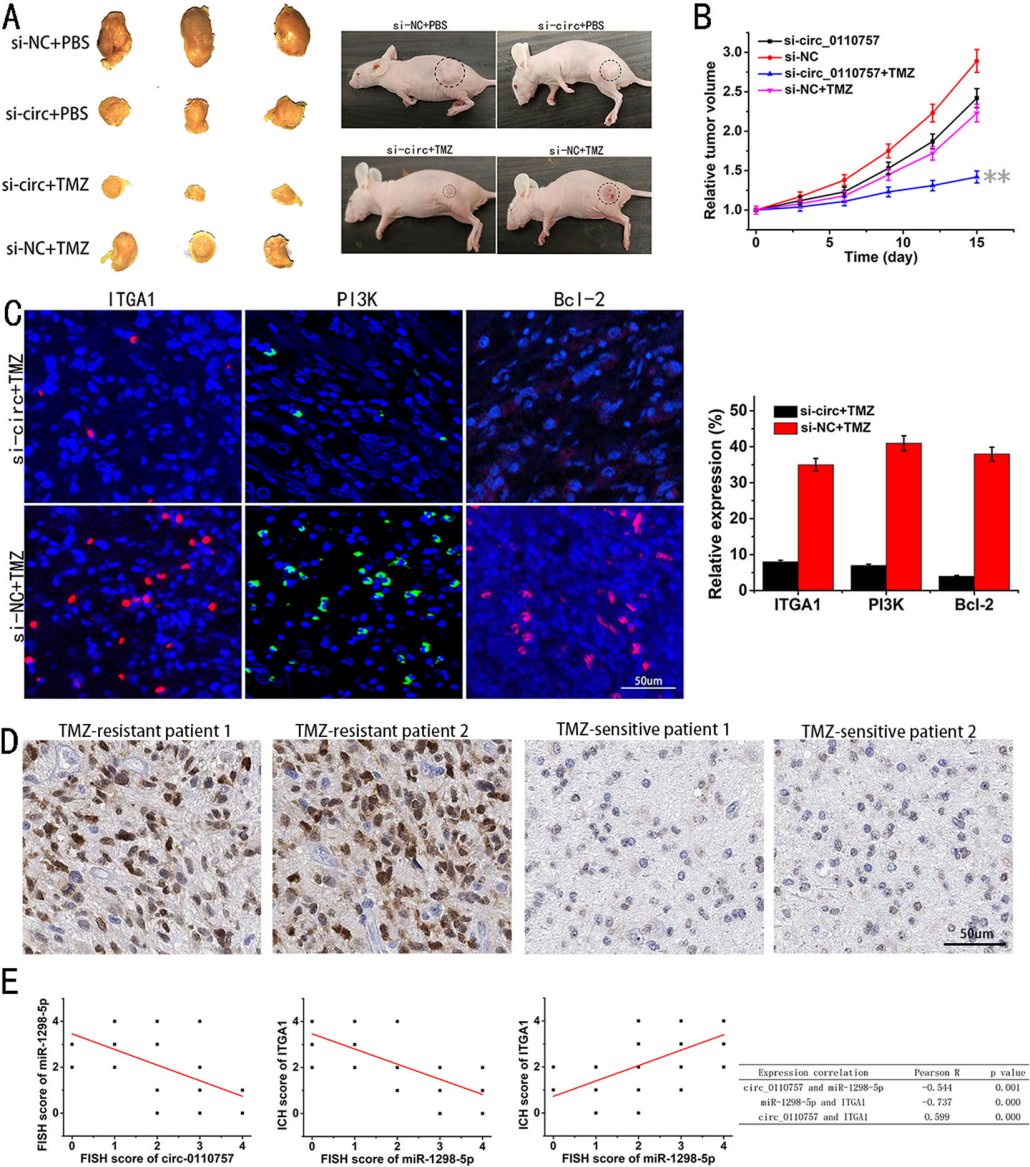
Hsa_circ_0110757 facilitates TMZ resistance of glioma cells in vivo. **A** U87/R cell xenograft tumors of nude mice with or without hsa_circ_0110757 knockdown after TMZ application at the end of the experiment. **B** The relative tumor volume for 15 days. **C** ITGA1 (red), PI3K (green), and Bcl-2 (red) in xenograft tumors were stained by immunofluorescent staining. **D** ITGA1 in TMZ-resistant or TMZ-sensitive glioma tissues from patients were stained by Immunohistochemical staining. **E** Correlations between hsa_circ_0110757 and miR-1298-5p expression levels and ITGA1 protein levels in 13 TMZ-resistant and 19 TMZ-sensitive glioma patients tissues. **P < 0.01.

**Fig. 7:**
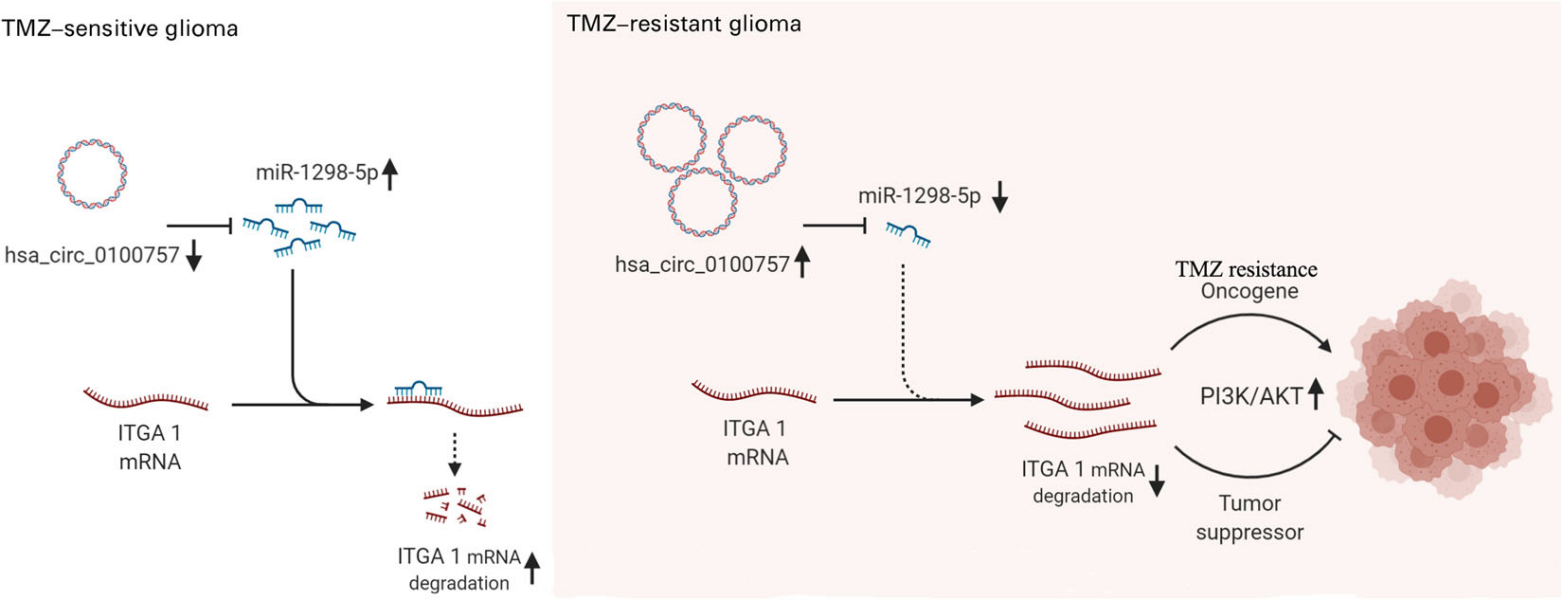
The mechanism diagram of hsa_circ_0110757 inducing TMZ resistance. Hsa_circ_0110757 is overexpressed in TMZ-resistant glioma and can effectively sponge hsa-miR-1298-5p to increase the expression of ITGA1. ITGA1 excites the PI3K/AKT signaling pathway, inhibits apoptosis and promotes TMZ resistance in glioma cells.

To study the effect of hsa_circ_0110757 in vivo, U87/R cells with or without hsa_circ_0110757 knockdown were subcutaneously injected into BALB/c nude mice. As shown in Fig. 6A, B, downregulated hsa_circ_0110757 in U87/R cells obviously reduced tumor growth and resensitized cells to TMZ. The immunofluorescence results indicated that the expression of ITGA1, PI3K, and Bcl-2 was significantly decreased with hsa_circ_0110757 inhibition (Fig. 6C). Moreover, compared with TMZ-sensitive glioma tissues, immunohistochemical staining showed that ITGA1 was significantly elevated in TMZ-resistant glioma tissues (Fig. 6D). In addition, the correlations among hsa_circ_0110757, miR-1298-5p, and the ITGA1 protein levels were recognized in 32 glioma samples (Fig. 6E). Based on the above results, as shown in Fig. 7, it was concluded that hsa_circ_0110757 facilitates the resistance of glioma cells to TMZ by targeting ITGA1 through hsa-miR-1298-5p.

## Discussion

TMZ is an alkane antitumor drug that does not need to go through liver activation and metabolism and is widely distributed throughout the body^20^. It can pass through the BBB and is widely used for the treatment of glioma^21^. However, long-term application is likely to induce acquired drug resistance, and drug resistance is still one of the problems that cannot be ignored in the treatment of tumors^22^. In high-throughput sequencing results, we found that hsa_circ_0110757 of Mcl-1 was overexpressed in TMZ-resistant glioma patient tissues and cells. Furthermore, compared with other circRNAs, hsa_circ_0110757 was obviously upregulated in TMZ-resistant glioma patients and may play a larger role in glioma. In this study, inhibition of hsa_circ_0110757 reduced the viability of U87/R cells and the number of invading cells and promoted cell apoptosis upon TMZ application.

miRNAs are endogenous noncoding RNAs with regulatory functions that can recognize target mRNAs by base complementary pairing and then degrade target mRNAs or inhibit the translation of target mRNAs^23^. Abnormal miRNA expression is related to the occurrence and development of tumors and can be used as both proto-oncogenes and tumor suppressor genes and is an important regulatory factor for the occurrence and development of tumors^24^. To identify miRNAs that hsa_circ_0110757 could sponge in glioma, eight miRNAs were chosen by overlapping the prediction results using miRanda, PITA, and RNAhybrid. Pull-down, PCR, and FISH assays indicated that hsa_circ_0110757 plays a role by sponging hsa-miR-1298-5p. Further experiments revealed that ITGA1 was the direct target of hsa-miR-1298-5p.

ITGA1, also known as CD49a or VLA1, encodes the α subunit of integrin receptors and plays an important role in cell–cell adhesion^25^. ITGA1 was highly expressed in TMZ-resistant pancreatic cancer, and downregulated ITGA1 restored the sensitivity of the above cells to TMZ^26^. According to the KEGG pathway database (www.kegg.jp/kegg/pathway.html), ITGA plays an important role in apoptosis by the PI3K/AKT pathway. In this study, the ITGA1 mRNA and protein levels were much higher in TMZ-resistant U87 cells than in TMZ-sensitive cells. Furthermore, hsa-miR-1298-5p mimics obviously decreased the expression of ITGA1, reduced cell viability, and induced apoptosis of U87/R cells.

According to the above results, a series of experiments proved that hsa_circ_0110757 weakened TMZ-induced apoptosis by activating PI3K/AKT, resulting in resistance to TMZ application. At the molecular level, hsa_circ_0110757 can sponge hsa-miR-1298-5p to eliminate the suppressive effect of hsa-miR-1298-5p on ITGA1, which then excites the PI3K/AKT signaling pathway and inhibits apoptosis in glioma cells. It was reported that upregulation of the PI3K/AKT pathway induced the upregulation of the antiapoptotic protein Bcl-2 and resulted in castration-resistant prostate cancer^27^. In this study, hsa_circ_0110757 may strengthen TMZ resistance by the PI3K/AKT pathway in glioma.

In summary, we found that hsa_circ_0110757 is overexpressed in TMZ-resistant glioma tissues and cells and can effectively sponge hsa-miR-1298-5p to increase the expression of ITGA1. It was also indicated that downregulation of hsa_circ_0110757 can significantly enhance TMZ sensitivity by regulating hsa-miR-1298-5p/ITGA1. This study demonstrates a novel theoretical basis that circRNAs exert function by sponging miRNAs and provides a new therapeutic strategy for glioma resistance.

## Materials and methods

### Glioma tissue samples

Seventeen TMZ-resistant and 17 TMZ-sensitive glioma samples were obtained from Xiangya Hospital Central South University. TMZ resistance was defined as tumor recurrence at the time of TMZ application, and TMZ sensitivity was defined as no tumor relapse at the time of TMZ application^28^. A total of 5 TMZ-resistant and 5 TMZ-sensitive glioma tissues were used for high-throughput sequencing, and 12 TMZ-resistant and 12 TMZ-sensitive glioma tissues were used to quantify ITGA1.

### Culture of resistant cells

The human glioma cell line U87 was acquired from the Advanced Research Center of Central South University. U87 cells were cultured in 1640 medium (HyClone, USA). Fetal bovine serum (HyClone, USA) was diluted to a concentration of 10% in 1640 medium, and penicillin–streptomycin (HyClone, USA) was added at a concentration of 1%. U87 cells were cultured in an incubator with 5% CO_2_ at 37 °C. To obtain TMZ (Sigma, 76899, USA) resistant cell lines, we gradually increased the exposure of U87 cells from 5 to 100 μmol/l of TMZ. The TMZ-resistant U87 cells were named U87/R cells^29^.

### Preparation of RNA, stability of circRNA, and PCR assay

Total RNA was extracted with TRIzol reagent (Invitrogen, USA) from glioma tissues and cells. The stability of circRNA was determined by RNase R (3 U/mg) treatment for 15 min at 37 °C. cDNAs were synthesized by a transcriptor-stranded synthesis kit (Takara, China). The mRNA expression of Mcl-1, ITGA1, hsa_circ_0110757, and hsa-miR-1298-5p was amplified by the ABI 7500 system (Applied Biosystems, USA). The primers used in this study are shown in Table S1.

### Cell transfection

For the construction of hsa_circ_0110757 overexpression plasmids, hsa_circ_0110757 cDNA was inserted into the PcDNA3.1 vector (GENECHEM, Shanghai, China). The PcDNA3.1 vector contains a front circular frame and a back circular frame. Lipofectamine 2000 (Beyotime Biotechnology, China) was used in transfection according to the manufacturer’s instructions. The luciferase reporter containing the hsa_circ_0110757 sequence in the 3′-UTR was constructed by subcloning the hsa_circ_0110757 fragment into the region directly downstream of a cytomegalovirus promoter-driven firefly luciferase cassette in a PcDNA3.1 vector. Mutations of each miRNA-binding site in the hsa_circ_0110757 sequence were created using a site-directed Mutagenesis Kit (Sangon Biotech, Shanghai, China). The mutations were introduced in both the hsa_circ_0110757-expressing vector and the luciferase reporter containing the hsa_circ_0110757 sequence.

### Cell viability

A total of 1 × 10^4^ cells per well were cultured in a 96-well plate for 24 h. Different concentrations of TMZ (0–600 μmol/l) were incubated with cells for 24 h. Then, the supernatant was removed and 100 μl MTT reagent (0.5 mg/ml) was added for 4 h. After removing the supernatant, 200 μl DMSO was added to dissolve purple crystals. A microporous plate detector (EnSpire 2300, PerkinElmer, USA) was used to detect the absorbance at 570 nm.

### Cell invasion assay

Cell invasion assay was carried out using 24-well transwells (8 μm, Corning, USA) coated with Matrigel (BD, USA). Overall, 1 × 105 cells in 500 μl DMEM (1% FBS) were added to the upper chamber, then 750 μl DMEM (10% FBS) was added in the lower chamber. After incubation for 48 h, Matrigel and cells in the upper chamber were removed. Cells on the lower surface of the membrane were fixed in 4% paraformaldehyde and stained with 0.5% crystal violet. Cells in five microscopic fields were counted and photographed.

### Annexin V-FITC/PI staining

The apoptosis rate was detected using the Annexin V-FITC/PI Apoptosis Detection kit (Beyotime Biotechnology, China). Overall, 5 × 105 cells were added into six-well plates. After treatment, cells were collected, washed with PBS, and resuspended in 0.5 ml staining buffer. Then, 5 μl Annexin V-FITC and 5 μl PI were added to the buffer and incubated at 37 °C for 15 min in the dark. Cells were analyzed by flow cytometry (BD, USA).

### RNA pull-down assay

As previously mentioned^30,31^, a biotin-labeled hsa_circ_0110757 probe was obtained from GeneChem (China). In short, 1 × 10^7^ hsa_circ_0110757-overexpressing U87 cells were lysed and incubated with hsa_circ_0110757 or oligo probe, and the RNA complex bound to the surface of beads were eluted anterior to RT-PCR. The probes sequences are shown in Table S2.

### Luciferase reporter gene assay

The wt 3′ UTR fragment of hsa_circ_0110757 was amplified and cloned into the pMIR-REPORT™ vector (GeneChem, China). The mutant (mt) hsa_circ_0110757 3′ UTR was induced by using a site-directed Mutagenesis Kit (Yeasen, China) to in hsa-miR-1298-5p binding sites. First, 6 × 10^4^ cells per well were incubated in 24-well plates for 24 h. hsa-miR-1298-5p or anti-hsa-miR-1298-5p with pMIR-REPORT-hsa_circ_0110757 (Wt) or pMIR-REPORT-hsa_circ_0110757 (Mut) was cotransfected into U87 cells by Lipofectamine 2000 (Invitrogen, USA) for 48 h. Finally, luciferase activity was detected by the dual-luciferase reporter assay system (Promega, USA).

### Western blot assay

Glioma tissues or cells were lysed by RIPA buffer (Beyotime Biotechnology, China) for 20 min. Total protein was collected from the cell lysate supernatant after centrifugation at 12,000 rpm for 10 min. The protein concentration was determined by a BCA Protein Assay Kit (Thermo Fisher, USA). Twenty micrograms of protein per lane was subjected to SDS-PAGE and then transferred to a PVDF membrane. The PVDF membrane was incubated with 5% skim milk for 2 h, followed by incubation with Mcl-1 primary antibody (Cell Signaling Technology, 39224, USA), ITGA1 primary antibody (Abnova, H00003672, USA), PI3K primary antibody (Abcam, ab140307, USA), p-AKT (phospho S473) primary antibody (Abcam, ab8932, USA), and Bcl-2 primary antibody (Abcam, ab32124, USA) for overnight. After washing with PBS, an HRP-conjugated secondary antibody (Beyotime Biotechnology, China) was incubated for 2 h. The signals of the PVDF membrane with chemiluminescence reagent were detected by chemiluminescence system (Bio-Rad, USA).

### Treatment of xenograft-bearing mice

A total of 10^7^ U87/R cells transfected with the si-hsa_circ_0110757 precursor were subcutaneously injected into BALB/c nude mice. When the tumor volume reached 100 mm^3^, TMZ (60 mg/kg) was applied intraperitoneally and tumor volume was measured every 2 days. After 15 days, the whole mice were systemically anesthetized and tumor tissues were collected for RT-PCR, western blot, and immunofluorescence assays.

### Statistical analysis

All data are shown as the mean ± standard deviation. SPSS 18.0 software was used to analyze significant differences. ANOVA and subsequent Tukey’s post hoc test were used to analyze the differences between groups. *P* < 0.05 was considered statistically significant.

## Supplementary information

Supplemental Information
