# Supplementary material for: Hsa_circ_0110757 upregulates ITGA1 to facilitate temozolomide resistance in glioma by suppressing hsa-miR-1298-5p
Source: Cell Death Dis. 2021 Mar 5;12(3):252. doi: 10.1038/s41419-021-03533-x (PMC7935991; doi:10.1038/s41419-021-03533-x)
Supplement: Supplementary file 1 — Supplemental Information [file 41419_2021_3533_MOESM1_ESM.docx]

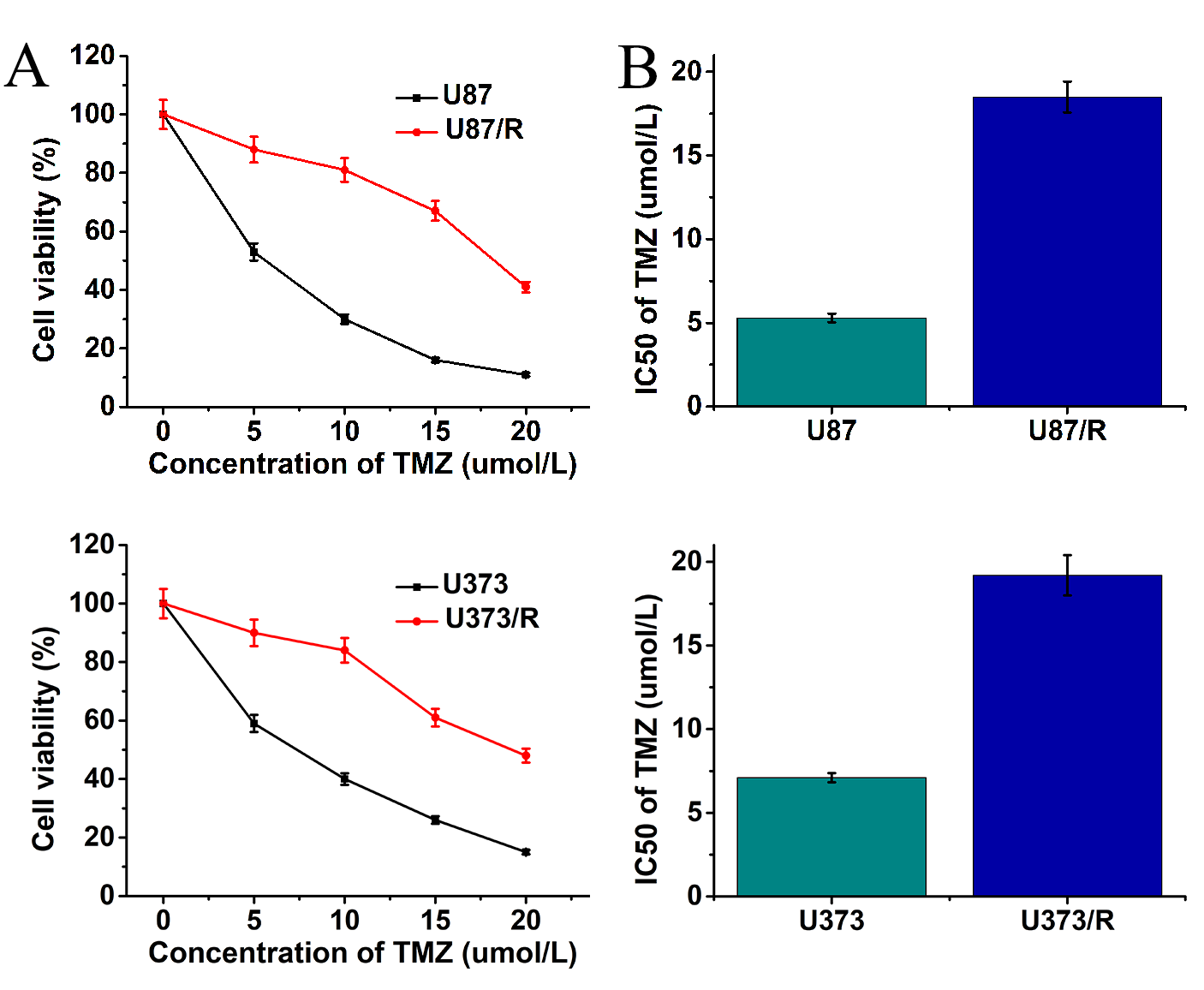


Fig. S1 (A) Cell viability of U87, U87/R, U373 and U373/R cells treated with different concentration of TMZ. (B) The IC50 of TMZ in U87, U87/R, U373 and U373/R cells.

Fig. S2 The specificity of siRNA 1 and siRNA 2 was validated using the hsa_circ_0110757 overexpressed U87 cells.


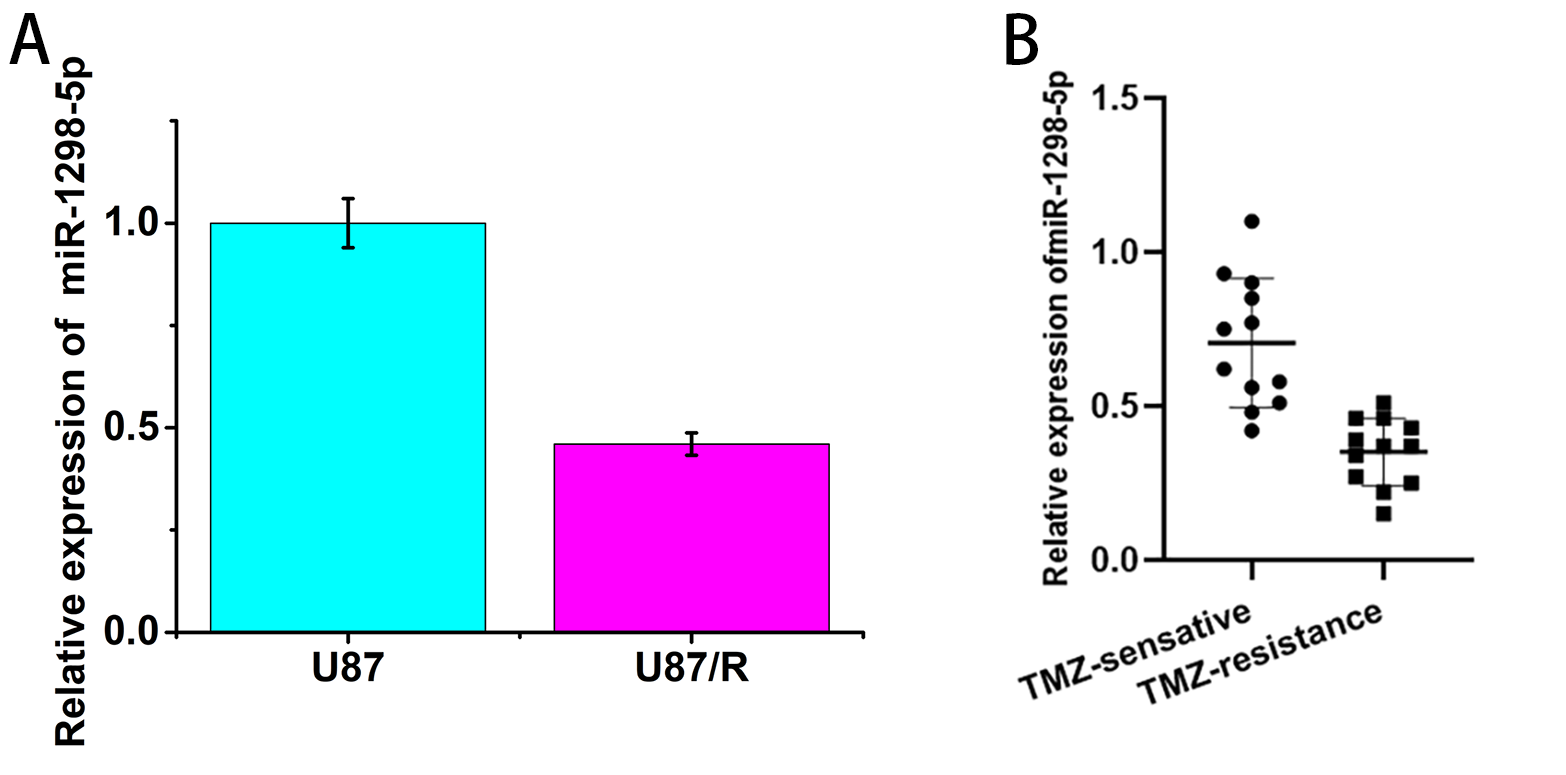


Fig. S3 The expression level of miR-1298-5p in U87 and U87/R cells (A), TMZ-sensitive and TMZ-resistant patients.


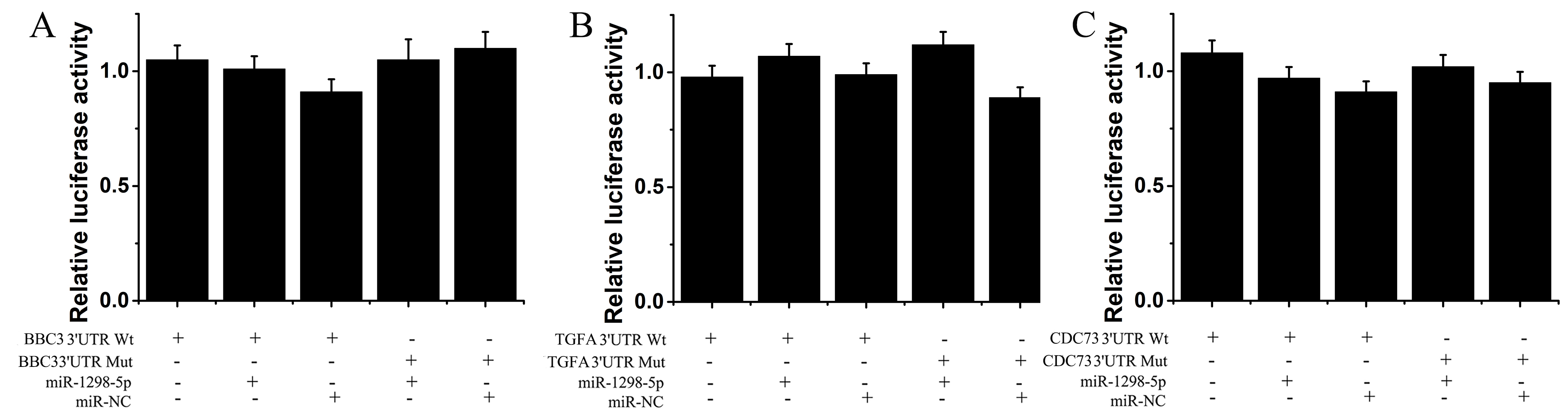


Fig. S4 The relative luciferase activities were analyzed in U87 cells cotransfected with miR-1298-5p or miR-NC and luciferase reporter vectors containing the WT or Mut 3’UTR of BBC3 (A), TGFA (B), and CDC73(C).

K

**Table S1** Primers and RNA sequences used in this study.

| **Primer sequence** |  |  |
| --- | --- | --- |
| hsa_circ_0110757 | Forward | TTTCTAGGATGGGTTTGTGGA |
|  | Reverse | CCGTGCGTCATAAAAACCTT |
| GAPDH | Forward | AATGGGCAGCCGTTAGGAAA |
|  | Reverse | GCGCCCAATACGACCAAATC |
| β-actin | Forward | CTCGCCTTTGCCGATCC |
|  | Reverse | TCTCCATGTCGTCCCAGTTG |
| ITGA1 | Forward | GGGAAGCTGCCAGTGAGATT |
|  | Reverse | GCAGCAGCGTAGAACAACAG |
| Mcl-1 | Reverse | GTGCCAGGAAGGGTTAGGAC |
|  | Forward | GGAAGGAAAAAGAGGCGTCG |
| hsa-miR-1298-5p | Forward | uucauucggcuguccagaugua |
| hsa-miR-331-3p | Forward | gccccugggccuauccuagaa |
| hsa-miR-515-5p | Forward | ttctccaaaagaaagcactttctg |
| hsa-miR-570 | Forward | aaagguaauugcaguuuuuccc |
| hsa-miR-651 | Forward | uuuaggauaagcuugacuuuug |
| hsa-miR-767-3p | Forward | ucugcucauaccccaugguuucu |
| hsa-miR-1224-3p | Forward | ccccaccuccucucuccucag |
| hsa-miR-1305 | Forward | uuuucaacucuaaugggagaga |

**Table S2 s**iRNA sequences used in this study.

| **siRNAs** |  |
| --- | --- |
| hsa_circ_0110757 si circ-1 | ACCTAGAAGGGGCCTTGGAGT |
| hsa_circ_0110757 si circ-2 | AGAAGGGGCCTTGGAGTGGAA |
| si ITGA1 | UCAUAUUCUGAAUCUCAUCCU |
| si nc | UUCUCCGAACGUGUCACGUTT |

**Table S3** miRNA mimics, and inhibitors sequences used in this study.

| **mimics and inhibitors** |  |
| --- | --- |
| mimics miR-1298-5p | uucauucggcuguccagaugua |
| mimics nc | UUCUCCGAACGUGUCACGUTT |
| anti-miR-1298-5p | UACAUCUGGACAGCCGAAUGAA |
| anti-nc | UUCUCCGAACGUGUCACGUTT |

**Table S4** Biotinylated probes sequences used in this study.

| **Biotinylated probes** |  |
| --- | --- |
| hsa_circ_0110757 | Biotin-CUUGGAGUGGAAGUAUGAAUGAAGCTCTGAGGACCGCACACG |
| Olige probe | Biotin-AAACAGTACTGGTGTGTAGTACGAGCTGAAGCTAC |

**Table S6** FISH probes sequences used in this study.

| **FISH probes** |  |
| --- | --- |
| hsa_circ_0110757 | Cy5-CUUGGAGUGGAAGUAUGAAUGAAGCTCTGA  GGACCGCACACG |
| anti-miR-1298-5p | Alexa 488-GAACCTACTCCCCTCTCTCCACC |
